# Supplementary material for: The Entero-Mammary Pathway and Perinatal Transmission of Gut Microbiota and SARS-CoV-2
Source: Int J Mol Sci. 2022 Sep 7;23(18):10306. doi: 10.3390/ijms231810306 (PMC9499685; doi:10.3390/ijms231810306)
Supplement: Supplementary file 1 [file ijms-23-10306-s001.zip › ijms-1884082-supplementary/Supplementary Material/Supplementary Materials.pdf]

International Journal of *Molecular Sciences*

MDPI

Article

The Entero-Mammary Pathway and Perinatal Transmission of Gut Microbiota and SARS-CoV-2

Carmen Josefina Juárez-Castelán, Juan Manuel Vélez-Ixta, Karina Corona-Cervantes, Alberto Piña-Escobedo, Yair Cruz-Narváez, Alejandro Hinojosa-Velasco, María Esther Landero-Montes de Oca, Eduardo Dávila-Gonzalez, Eduardo González-del Olmo, Fernando Guadalupe Bastida-González, Paola Berenice Zárate-Segura, Jaime García-Mena

Supplementary Material

Tables

Table S1. 5′ to 3′ sequence of barcoded primers for bacteria used in this study.

Primer	Ion Torrent linker	Golay barcode	Spacer	16S rRNA gene
V3-341 F1	CCATCTCATCCCTGCGTGTCTCCGACTCAG	GATCTGCGATCC	GT	CCTACGGGAGGCAGCAG
V3-341 F2	CCATCTCATCCCTGCGTGTCTCCGACTCAG	CAGCTCATCAGC	GT	CCTACGGGAGGCAGCAG
V3-341 F3	CCATCTCATCCCTGCGTGTCTCCGACTCAG	CAAACAACAGCT	GT	CCTACGGGAGGCAGCAG
V3-341 F4	CCATCTCATCCCTGCGTGTCTCCGACTCAG	GCAACACCATCC	GT	CCTACGGGAGGCAGCAG
V3-341 F5	CCATCTCATCCCTGCGTGTCTCCGACTCAG	GCGATATATCGC	GT	CCTACGGGAGGCAGCAG
V3-341 F6	CCATCTCATCCCTGCGTGTCTCCGACTCAG	CGAGCAATCCTA	GT	CCTACGGGAGGCAGCAG
V3-341 F7	CCATCTCATCCCTGCGTGTCTCCGACTCAG	AGTCGTGCACAT	GT	CCTACGGGAGGCAGCAG
V3-341 F8	CCATCTCATCCCTGCGTGTCTCCGACTCAG	GTATCTGCGCGT	GT	CCTACGGGAGGCAGCAG
V3-341 F9	CCATCTCATCCCTGCGTGTCTCCGACTCAG	CGAGGGAAAGTC	GT	CCTACGGGAGGCAGCAG
V3-341 F10	CCATCTCATCCCTGCGTGTCTCCGACTCAG	CAAATTCGGGAT	GT	CCTACGGGAGGCAGCAG
V3-341 F11	CCATCTCATCCCTGCGTGTCTCCGACTCAG	AGATTGACCAAC	GT	CCTACGGGAGGCAGCAG
V3-341 F12	CCATCTCATCCCTGCGTGTCTCCGACTCAG	AGTTACGAGCTA	GT	CCTACGGGAGGCAGCAG
V3-341 F13	CCATCTCATCCCTGCGTGTCTCCGACTCAG	GCATATGCACTG	GT	CCTACGGGAGGCAGCAG
V3-341 F14	CCATCTCATCCCTGCGTGTCTCCGACTCAG	CAACTCCCGTGA	GT	CCTACGGGAGGCAGCAG
V3-341 F15	CCATCTCATCCCTGCGTGTCTCCGACTCAG	TTGCGTTAGCAG	GT	CCTACGGGAGGCAGCAG
V3-341 F16	CCATCTCATCCCTGCGTGTCTCCGACTCAG	TACGAGCCCTAA	GT	CCTACGGGAGGCAGCAG
V3-341 F17	CCATCTCATCCCTGCGTGTCTCCGACTCAG	CACTACGCTAGA	GT	CCTACGGGAGGCAGCAG
V3-341 F18	CCATCTCATCCCTGCGTGTCTCCGACTCAG	TGCAGTCCTCGA	GT	CCTACGGGAGGCAGCAG
V3-341 F19	CCATCTCATCCCTGCGTGTCTCCGACTCAG	ACCATAGCTCCG	GT	CCTACGGGAGGCAGCAG
V3-341 F20	CCATCTCATCCCTGCGTGTCTCCGACTCAG	TGGACATCTCTT	GT	CCTACGGGAGGCAGCAG
V3-341 F21	CCATCTCATCCCTGCGTGTCTCCGACTCAG	GAACACTTTGGA	GT	CCTACGGGAGGCAGCAG
V3-341 F22	CCATCTCATCCCTGCGTGTCTCCGACTCAG	GAGCCATCTGTA	GT	CCTACGGGAGGCAGCAG
V3-341 F23	CCATCTCATCCCTGCGTGTCTCCGACTCAG	TTGGGTACACGT	GT	CCTACGGGAGGCAGCAG
V3-341 F24	CCATCTCATCCCTGCGTGTCTCCGACTCAG	AAGGCGCTCCTT	GT	CCTACGGGAGGCAGCAG
V3-341 F25	CCATCTCATCCCTGCGTGTCTCCGACTCAG	TAATACGGATCG	GT	CCTACGGGAGGCAGCAG
V3-341 F26	CCATCTCATCCCTGCGTGTCTCCGACTCAG	TCGGAATTAGAC	GT	CCTACGGGAGGCAGCAG
V3-341 F27	CCATCTCATCCCTGCGTGTCTCCGACTCAG	TGTGAATTCGGA	GT	CCTACGGGAGGCAGCAG
V3-341 F28	CCATCTCATCCCTGCGTGTCTCCGACTCAG	CATTCGTGGCGT	GT	CCTACGGGAGGCAGCAG
V3-341 F29	CCATCTCATCCCTGCGTGTCTCCGACTCAG	AACGCACGCTAG	GT	CCTACGGGAGGCAGCAG
V3-341 F30	CCATCTCATCCCTGCGTGTCTCCGACTCAG	ACACTGTTCATG	GT	CCTACGGGAGGCAGCAG
V3-341 F31	CCATCTCATCCCTGCGTGTCTCCGACTCAG	ACCAGACGATGC	GT	CCTACGGGAGGCAGCAG
V3-341 F32	CCATCTCATCCCTGCGTGTCTCCGACTCAG	ACGCTCATGGAT	GT	CCTACGGGAGGCAGCAG
V3-341 F33	CCATCTCATCCCTGCGTGTCTCCGACTCAG	ACTCACGGTATG	GT	CCTACGGGAGGCAGCAG
V3-341 F34	CCATCTCATCCCTGCGTGTCTCCGACTCAG	AGACCGTCAGAC	GT	CCTACGGGAGGCAGCAG
V3-341 F35	CCATCTCATCCCTGCGTGTCTCCGACTCAG	AGCACGAGCCTA	GT	CCTACGGGAGGCAGCAG
V3-341 F36	CCATCTCATCCCTGCGTGTCTCCGACTCAG	ACAGACCACTCA	GT	CCTACGGGAGGCAGCAG
V3-341 F37	CCATCTCATCCCTGCGTGTCTCCGACTCAG	ACCAGCGACTAG	GT	CCTACGGGAGGCAGCAG
V3-341 F38	CCATCTCATCCCTGCGTGTCTCCGACTCAG	ACGGATCGTCAG	GT	CCTACGGGAGGCAGCAG
V3-341 F39	CCATCTCATCCCTGCGTGTCTCCGACTCAG	AGCTTGACAGCT	GT	CCTACGGGAGGCAGCAG
V3-341 F40	CCATCTCATCCCTGCGTGTCTCCGACTCAG	AACTGTGCGTAC	GT	CCTACGGGAGGCAGCAG
V3-341 F41	CCATCTCATCCCTGCGTGTCTCCGACTCAG	ACCGCAGAGTCA	GT	CCTACGGGAGGCAGCAG
V3-341 F42	CCATCTCATCCCTGCGTGTCTCCGACTCAG	ACGGTGAGTGTC	GT	CCTACGGGAGGCAGCAG

V3-341 F43	CCATCTCATCCCTGCGTGTCTCCGACTCAG	ACTCGATTCGAT	GT	CCTACGGGAGGCAGCAG
V3-341 F44	CCATCTCATCCCTGCGTGTCTCCGACTCAG	AGACTGCGTACT	GT	CCTACGGGAGGCAGCAG
V3-341 F45	CCATCTCATCCCTGCGTGTCTCCGACTCAG	AGCAGTCGCGAT	GT	CCTACGGGAGGCAGCAG
V3-341 F46	CCATCTCATCCCTGCGTGTCTCCGACTCAG	AGGACGCACTGT	GT	CCTACGGGAGGCAGCAG
V3-341 F47	CCATCTCATCCCTGCGTGTCTCCGACTCAG	AAGAGATGTCGA	GT	CCTACGGGAGGCAGCAG
V3-341 F48	CCATCTCATCCCTGCGTGTCTCCGACTCAG	ACAGCAGTGGTC	GT	CCTACGGGAGGCAGCAG
V3-341 F49	CCATCTCATCCCTGCGTGTCTCCGACTCAG	ACGTACTCAGTG	GT	CCTACGGGAGGCAGCAG
V3-341 F50	CCATCTCATCCCTGCGTGTCTCCGACTCAG	ACTCGCACAGGA	GT	CCTACGGGAGGCAGCAG
V3-341 F51	CCATCTCATCCCTGCGTGTCTCCGACTCAG	AGAGAGCAAGTG	GT	CCTACGGGAGGCAGCAG
V3-341 F52	CCATCTCATCCCTGCGTGTCTCCGACTCAG	AGCATATGAGAG	GT	CCTACGGGAGGCAGCAG
V3-341 F53	CCATCTCATCCCTGCGTGTCTCCGACTCAG	AGGCTACACGAC	GT	CCTACGGGAGGCAGCAG
V3-341 F54	CCATCTCATCCCTGCGTGTCTCCGACTCAG	AAGCTGCAGTCG	GT	CCTACGGGAGGCAGCAG
V3-341 F55	CCATCTCATCCCTGCGTGTCTCCGACTCAG	ACAGCTAGCTTG	GT	CCTACGGGAGGCAGCAG
V3-341 F56	CCATCTCATCCCTGCGTGTCTCCGACTCAG	ACCTGTCTCTCT	GT	CCTACGGGAGGCAGCAG
V3-341 F57	CCATCTCATCCCTGCGTGTCTCCGACTCAG	ACGTCTGTAGCA	GT	CCTACGGGAGGCAGCAG
V3-341 F58	CCATCTCATCCCTGCGTGTCTCCGACTCAG	AGAGCAAGAGCA	GT	CCTACGGGAGGCAGCAG
V3-341 F59	CCATCTCATCCCTGCGTGTCTCCGACTCAG	AGCCATACTGAC	GT	CCTACGGGAGGCAGCAG
V3-341 F60	CCATCTCATCCCTGCGTGTCTCCGACTCAG	AGGTGTGATCGC	GT	CCTACGGGAGGCAGCAG
V3-341 F61	CCATCTCATCCCTGCGTGTCTCCGACTCAG	AATCAGTCTCGT	GT	CCTACGGGAGGCAGCAG
V3-341 F62	CCATCTCATCCCTGCGTGTCTCCGACTCAG	ACGACGTCTTAG	GT	CCTACGGGAGGCAGCAG
V3-341 F63	CCATCTCATCCCTGCGTGTCTCCGACTCAG	ACGTGAGAGAAT	GT	CCTACGGGAGGCAGCAG
V3-341 F64	CCATCTCATCCCTGCGTGTCTCCGACTCAG	ACTGACAGCCAT	GT	CCTACGGGAGGCAGCAG
V3-341 F65	CCATCTCATCCCTGCGTGTCTCCGACTCAG	AGCGACTGTGCA	GT	CCTACGGGAGGCAGCAG
V3-341 F66	CCATCTCATCCCTGCGTGTCTCCGACTCAG	AGTACGCTCGAG	GT	CCTACGGGAGGCAGCAG
V3-341 F67	CCATCTCATCCCTGCGTGTCTCCGACTCAG	AATCGTGACTCG	GT	CCTACGGGAGGCAGCAG
V3-341 F68	CCATCTCATCCCTGCGTGTCTCCGACTCAG	ACGAGTGCTATC	GT	CCTACGGGAGGCAGCAG
V3-341 F69	CCATCTCATCCCTGCGTGTCTCCGACTCAG	ACTGATCCTAGT	GT	CCTACGGGAGGCAGCAG
V3-341 F70	CCATCTCATCCCTGCGTGTCTCCGACTCAG	AGAGTCCTGAGC	GT	CCTACGGGAGGCAGCAG
V3-341 F71	CCATCTCATCCCTGCGTGTCTCCGACTCAG	AGCGAGCTATCT	GT	CCTACGGGAGGCAGCAG
V3-341 F72	CCATCTCATCCCTGCGTGTCTCCGACTCAG	AGTACTGCAGGC	GT	CCTACGGGAGGCAGCAG
V3-341 F73	CCATCTCATCCCTGCGTGTCTCCGACTCAG	ACACACTATGGC	GT	CCTACGGGAGGCAGCAG
V3-341 F74	CCATCTCATCCCTGCGTGTCTCCGACTCAG	ACGATGCGACCA	GT	CCTACGGGAGGCAGCAG
V3-341 F75	CCATCTCATCCCTGCGTGTCTCCGACTCAG	ACGTTAGCACAC	GT	CCTACGGGAGGCAGCAG
V3-341 F76	CCATCTCATCCCTGCGTGTCTCCGACTCAG	ACTGTACGCGTA	GT	CCTACGGGAGGCAGCAG
V3-341 F77	CCATCTCATCCCTGCGTGTCTCCGACTCAG	AGATACACGCGC	GT	CCTACGGGAGGCAGCAG
V3-341 F78	CCATCTCATCCCTGCGTGTCTCCGACTCAG	AGCGCTGATGTG	GT	CCTACGGGAGGCAGCAG
V3-341 F79	CCATCTCATCCCTGCGTGTCTCCGACTCAG	ACACATGTCTAC	GT	CCTACGGGAGGCAGCAG
V3-341 F80	CCATCTCATCCCTGCGTGTCTCCGACTCAG	ACATGATCGTTC	GT	CCTACGGGAGGCAGCAG
V3-341 F81	CCATCTCATCCCTGCGTGTCTCCGACTCAG	ACGCAACTGCTA	GT	CCTACGGGAGGCAGCAG
V3-341 F82	CCATCTCATCCCTGCGTGTCTCCGACTCAG	ACTGTCGAAGCT	GT	CCTACGGGAGGCAGCAG
V3-341 F83	CCATCTCATCCCTGCGTGTCTCCGACTCAG	AGCGTAGGTCGT	GT	CCTACGGGAGGCAGCAG
V3-341 F84	CCATCTCATCCCTGCGTGTCTCCGACTCAG	ACACGAGCCACA	GT	CCTACGGGAGGCAGCAG
V3-341 F85	CCATCTCATCCCTGCGTGTCTCCGACTCAG	ACATGTCACGTG	GT	CCTACGGGAGGCAGCAG
V3-341 F86	CCATCTCATCCCTGCGTGTCTCCGACTCAG	ACGCGATACTGG	GT	CCTACGGGAGGCAGCAG
V3-341 F87	CCATCTCATCCCTGCGTGTCTCCGACTCAG	ACTACGTGTGGT	GT	CCTACGGGAGGCAGCAG
V3-341 F88	CCATCTCATCCCTGCGTGTCTCCGACTCAG	ACTGTGACTTCA	GT	CCTACGGGAGGCAGCAG
V3-341 F89	CCATCTCATCCCTGCGTGTCTCCGACTCAG	AGATCTCTGCAT	GT	CCTACGGGAGGCAGCAG
V3-341 F90	CCATCTCATCCCTGCGTGTCTCCGACTCAG	AGCTATCCACGA	GT	CCTACGGGAGGCAGCAG
V3-341 F91	CCATCTCATCCCTGCGTGTCTCCGACTCAG	AGTCCATAGCTG	GT	CCTACGGGAGGCAGCAG
V3-341 F92	CCATCTCATCCCTGCGTGTCTCCGACTCAG	ACACGGTGTCTA	GT	CCTACGGGAGGCAGCAG
V3-341 F93	CCATCTCATCCCTGCGTGTCTCCGACTCAG	ACATTCAGCGCA	GT	CCTACGGGAGGCAGCAG
V3-341 F94	CCATCTCATCCCTGCGTGTCTCCGACTCAG	ACTTGTAGCAGC	GT	CCTACGGGAGGCAGCAG
V3-341 F95	CCATCTCATCCCTGCGTGTCTCCGACTCAG	AGATGTTCTGCT	GT	CCTACGGGAGGCAGCAG
V3-341 F96	CCATCTCATCCCTGCGTGTCTCCGACTCAG	AGCTCCATACAG	GT	CCTACGGGAGGCAGCAG
V3-341 F97	CCATCTCATCCCTGCGTGTCTCCGACTCAG	ACGCTATCTGGA	GT	CCTACGGGAGGCAGCAG
V3-341 F98	CCATCTCATCCCTGCGTGTCTCCGACTCAG	ACTATTGTCACG	GT	CCTACGGGAGGCAGCAG
V3-341 F99	CCATCTCATCCCTGCGTGTCTCCGACTCAG	AGAACACGTCTC	GT	CCTACGGGAGGCAGCAG
V3-341 F100	CCATCTCATCCCTGCGTGTCTCCGACTCAG	AGTGAGAGAAGC	GT	CCTACGGGAGGCAGCAG
V3-518 R	CCTCTCTATGGGCAGTCGGTGAT	---------------------------	GT	ATTACCGCGGCTGCTGG

V3-341F forward primer is complementary to positions 340-356, while V3-518R reverse primer is complementary to positions 517-533 of the *Escherichia coli* 16S rDNA molecule *rrnB* GenBank J01859.1. "---------------------------" indicates no sequence.

International Journal of *Molecular Sciences*

MDPI

Table S2. Relative abundance at phylum level in MRS, HC, and NRS samples grouped according to SARS-CoV-2 rectal swab RT-ddPCR detection.

Phylum	MRS (% ± SD)		p*	HC (% ± SD)		p*	NRS (% ± SD)		p*
	Negative	Positive		Negative	Positive		Negative	Positive	
n	28	17	--	28	21	--	21	24	--
Actinobacteriota	5.07±5.35	7.41±11.0	0.344	9.63±6.83	13.48±11.56	0.184	10.05±11.19	13.67±13.90	0.338
Bacteroidota	34.02±26.31	32.36±23.03	0.831	3.52±8.23	5.80±13.78	0.504	3.23±9.88	4.72±10.27	0.624
Firmicutes	39.91±18.73	44.64±19.02	0.420	64.41±28.76	57.41±27.61	0.392	44.26±36.09	38.65±30.27	0.578
Proteobacteria	18.92±23.47	13.44±19.53	0.629	17.46±18.56	20.70±25.11	0.628	41.19±34.76	41.53±34.53	0.759
Others	1.96±3.50	1.52±2.56	0.426	1.38±1.85	1.72±2.80	0.622	0.78±1.33	0.91±1.55	0.973

MRS, Mother Rectal Swab; HC, Human Colostrum; NRS, Neonate Rectal Swab; %, Relative abundance of each taxon; SD, Standard Deviation; **p*-value was calculated according to T-test.

Table S3. Relative abundance at family level in MRS samples grouped according to SARS-CoV-2 rectal swab RT-ddPCR detection.

Phylum	Family	Negative			Positive			p**
		% ± SD	CV	Range*	% ± SD	CV	Range*	
Actinobacteriota	Bifidobacteriaceae	0.8±0.81	1.02	0.0-2.9	5.62±10.89	2.07	0.0-45.6	0.040
	Corynebacteriaceae	0.07±0.10	1.35	0.0-0.3	0.025±0.11	4.57	0.0-0.6	0.128
	Microbacteriaceae	1.38±2.12	1.54	0.0-7.0	0.25±0.31	1.26	0.0-1.2	0.044
	Propionibacteriaceae	0.06±0.11	1.72	0.0-0.3	0.08±0.32	3.76	0.0-1.7	0.754
Bacteroidota	Bacteroidaceae	6.26±12.54	2.00	0.0-45.4	12.98±9.84	1.22	0.0-49.2	0.123
	Porphyromonadaceae	3.28±7.60	2.31	0.0-30.3	0.61±9.84	4.82	0.0-15.7	0.182
	Prevotellaceae	20.88±21.57	1.03	0.2-63.3	13.02±19.82	1.52	0.0-81.2	0.230
	Tannerellaceae	1.04±1.79	1.71	0.0-6.6	2.96±7.05	2.37	0.0-37.8	0.180
Firmicutes	Clostridiaceae	1.73±2.98	1.71	0.0-9.0	0.51±1.08	2.10	0.0-4.9	0.120
	Enterococcaceae	3.0±9.73	3.24	0.0-40.1	1.05±4.97	4.73	0.0-26.4	0.451
	Erysipelotrichaceae	2.6±4.06	1.56	0.0-14.1	1.35±1.88	1.39	0.0-6.2	0.247
	Lachnospiraceae	5.37±4.61	0.85	0.0-16.1	8.30±5.41	0.65	1.5-21.2	0.060
	Lactobacillaceae	0.41±0.58	1.43	0.0-2.0	0.32±0.62	1.95	0.0-2.2	0.630
	Oscillospiraceae	0.94±1.11	1.18	0.0-4.1	3.05±3.88	1.27	0.0-16.4	0.010
	Peptostreptococcacea-Tissierellales	2.74±5.58	2.03	0.0-18.2	0.50±2.47	4.90	0.0-13.1	0.133
	Ruminococcaceae	7.78±7.90	1.01	0.0-29.8	12.35±12.39	1.00	0.9-49.6	0.138
	Staphylococcaceae	0.38±1.24	3.25	0.0-5.1	0.06±0.24	4.04	0.0-1.3	0.307
	Streptococcaceae	1.63±1.96	1.20	0.0-6.1	0.78±0.70	0.90	0.0-2.3	0.101
	Veillonellaceae	5.16±8.99	1.74	0.0-31.2	9.27±12.23	1.31	0.0-43.5	0.203
Proteobacteria	Comamonadaceae	0.27±0.58	2.16	0.0-2.2	0.12±0.20	1.63	0.0-0.8	0.335
	Enterobacteriaceae	12.98±24.74	1.90	0.0-88.8	11.31±18.94	1.67	0.0-86.8	0.812
	Moraxellaceae	0.07±0.14	2.11	0.0-0.5	0.06±0.19	3.24	0.0-1.0	0.849
	Pseudomonadaceae	0.03±0.08	2.44	0.0-0.3	0.08±0.22	2.73	0.0-0.8	0.328
	Sphingomonadaceae	2.07±3.88	1.87	0.0-13.0	0.27±0.59	1.97	0.0-2.5	0.076
	Xanthobacteraceae	0.76±1.27	1.66	0.0-4.0	0.14±0.31	2.20	0.0-1.2	0.064
	Others	17.67±9.84	0.56	1.4-35.9	15.03±12.05	0.8	0.2-48.6	0.427

Positive (n=17); Negative (n=28); MRS, Mother Rectal Swab; %, Relative abundance of each taxon; SD, Standard Deviation; CV, Coefficient of Variation; *Range, denotes the minimum and maximum values in the relative abundance for each taxon; ***p*-value was calculated according to T-test; $p< 0.05$ are considered statistically significant differences.

International Journal of Molecular Sciences

MDPI

Table S4. Relative abundance at family level in HC samples grouped according to SARS-CoV-2 rectal swab RT-ddPCR detection.

Phylum	Family	Negative			Positive			
		% ± SD	CV	Range*	% ± SD	CV	Range*	*p***
Actinobacteriota	Bifidobacteriaceae	0.34±0.53	1.59	0.0-2.2	0.35±0.62	1.78	0.0-2.8	0.944
	Corynebacteriaceae	1.85±1.92	1.04	0.0-6.5	3.26±4.90	1.5	0.0-21.5	0.224
	Microbacteriaceae	1.7±2.31	1.36	0.0-9.1	1.9±2.45	1.29	0.0-8.8	0.778
	Propionibacteriaceae	2.57±2.71	1.05	0.1-9.3	4.58±4.60	1.01	0.0-17.8	0.085
Bacteroidota	Bacteroidaceae	0.07±0.22	3.23	0.0-1.1	0.06±0.16	2.53	0.0-0.6	0.912
	Porphyromonadaceae	0.05±0.21	3.98	0.0-1.1	1.2±5.16	4.3	0.0-23.7	0.320
	Prevotellaceae	2.73±8.04	2.95	0.0-41	3.80±13.07	3.44	0.0-60.4	0.741
	Tannerellaceae	0.00±0.00	0.0	0.0-0.0	0.01±0.04	4.58	0.0-0.2	0.329
Firmicutes	Clostridiaceae	0.51±0.63	1.23	0.0-2.2	0.034±0.38	1.11	0.0-1.2	0.252
	Enterococcaceae	0.15±0.39	2.68	0.0-2.0	0.37±0.95	2.60	0.0-4.3	0.328
	Erysipelotrichaceae	0.13±0.34	1.3	0.0-1.3	0.13±0.28	2.16	0.0-1.1	1.000
	Lachnospiraceae	0.73±1.14	1.57	0.0-4.9	1.24±2.67	2.15	0.0-11.8	0.419
	Lactobacillaceae	2.4±3.24	1.35	0.0-12.8	6.44±18.11	2.81	0.0-78.5	0.323
	Oscillospiraceae	0.8±0.14	1.74	0.0-0.5	0.16±0.46	2.93	0.0-2.1	0.456
	Peptostreptococcacea-Tissierellales	0.51±0.8	1.56	0.0-2.8	1.31±2.87	2.19	0.0-11.8	0.227
	Ruminococcaceae	0.4±0.52	1.31	0.0-1.7	0.64±1.16	1.82	0.0-4.5	0.382
	Staphylococcaceae	55.27±34.92	0.63	1.2-99.0	40.58±31.46	0.78	0.0-99.8	0.129
	Streptococcaceae	2.13±4.5	2.11	0.0-18.4	4.10±8.61	2.10	0.0-33.5	0.345
	Veillonellaceae	1.21±5.05	4.18	0.0-26.9	1.15±2.34	2.04	0.0-8.7	0.956
Proteobacteria	Comamonadaceae	1.2±2.23	1.85	0.0-11.4	1.05±1.77	1.69	0.0-6.9	0.792
	Enterobacteriaceae	2.29±4.6	2.0	0.0-21.1	5.93±21.61	3.65	0.0-100	0.456
	Moraxellaceae	3.1±3.89	1.25	0.0-16.6	2.76±3.08	1.12	0.0-11.1	0.733
	Pseudomonadaceae	0.92±1.85	2.0	0.0-8.2	3.4±10.12	2.97	0.0-45.2	0.279
	Sphingomonadaceae	1.45±1.77	1.22	0.0-6.8	1.93±2.37	1.22	0.0-9.8	0.437
	Xanthobacteraceae	1.45±2.94	2.04	0.0-14.7	1.62±2.01	1.24	0.0-7.0	0.803
	Others	13.03±14.77	1.13	0.0-58.1	10.74±10.29	0.96	0.0-34.8	0.527

Positive (n=21), Negative (n=28), HC, Human Colostrum; %, Relative abundance of each taxon; SD, Standard Deviation; CV, Coefficient of Variation; *Range, denotes the minimum and maximum values in the relative abundance for each taxon; ***p*-value was calculated according to T-test, $p < 0.05$ are considered statistically significant differences.

International Journal of
Molecular Sciences

MDPI

Table S5. Relative abundance at family level in NRS samples grouped according to SARS-CoV-2 rectal swab RT-ddPCR detection.

Phylum	Family	Negative			Positive			
		% ± SD	CV	Range*	% ± SD	CV	Range*	*p***
Actinobacteriota	Bifidobacteriaceae	0.74±2.51	3.38	0.0-11.5	2.71±5.88	2.17	0.0-24.0	0.145
	Corynebacteriaceae	1.26±2.56	2.04	0.0-11.6	0.78±1.34	1.73	0.0.4.8	0.444
	Microbacteriaceae	3.24±4.09	1.26	0.0-10.8	2.94±5.05	1.72	0.0-16.5	0.826
	Propionibacteriaceae	2.61±4.13	1.58	0.0-14.7	3.23±7.26	2.25	0.0-33.8	0.721
Bacteroidota	Bacteroidaceae	0.92±3.46	3.75	0.0-15.8	1.31±4.54	3.46	0.0-22.2	0.746
	Porphyromonadaceae	0.02±0.06	2.62	0.0-0.2	0.04±0.12	2.82	0.0-0.4	0.521
	Prevotellaceae	1.51±5.34	3.54	0.0-24.6	1.23±2.54	2.06	0.0-12.1	0.830
	Tannerellaceae	0.14±0.59	4.26	0.0-2.7	0.12±0.37	3.07	0.0-1.7	0.908
Firmicutes	Clostridiaceae	4.28±8.51	1.99	0.0-29.5	5.41±10.85	2.00	0.0-35.7	0.696
	Enterococcaceae	0.75±1.4	1.87	0.0-5.4	2.7±6.41	2.38	0.0-23.8	0.159
	Erysipelotrichaceae	0.17±0.66	3.95	0.0-3.0	1.1±2.71	2.47	0.0-12.0	0.116
	Lachnospiraceae	1.19±2.91	2.45	0.0-12.9	5.85±11.72	2.00	0.0-48.1	0.070
	Lactobacillaceae	5.19±10.38	2.0	0.0-34.8	6.92±12.98	1.88	0.0-40.3	0.622
	Oscillospiraceae	0.03±0.06	1.96	0.0-0.2	0.44±1.02	2.29	0.0-4.4	0.057
	Peptostreptococcacea-Tissierellales	0.0±0.09	2.27	0.0-0.3	0.05±0.09	2.03	0.0-0.3	0.774
	Ruminococcaceae	0.68±1.55	2.29	0.0-6.9	3.83±6.58	1.72	0.0-25.0	0.031
	Staphylococcaceae	0.5±0.66	1.32	0.0-2.3	1.67±3.94	2.37	0.0-15.1	0.166
	Streptococcaceae	28.28±39.17	1.39	0.0-97.6	5.03±10.57	2.10	0.0-49.4	0.014
	Veillonellaceae	2.41±6.67	2.76	0.0-28.4	3.49±9.71	2.78	0.0-46.0	0.664
Proteobacteria	Comamonadaceae	3.59±5.68	1.58	0.0-22.3	2.48±5.51	2.23	0.0-26.6	0.510
	Enterobacteriaceae	20.37±34.19	1.68	0.0-99.9	25.27±36.21	1.43	0.0-100.0	0.643
	Moraxellaceae	1.24±2.01	1.62	0.0-8.6	1.96±2.57	1.31	0.0-7.9	0.301
	Pseudomonadaceae	4.20±18.72	4.45	0.0-85.9	0.3±0.48	1.59	0.0-1.7	0.351
	Sphingomonadaceae	2.72±3.35	1.23	0.0-9.0	4.07±6.58	1.62	0.0-19.0	0.384
	Xanthobacteraceae	4.87±9.55	1.96	0.0-28.3	2.61±4.94	1.89	0.0-20.2	0.337
	Others	8.55±8.32	0.97	0.0-23.7	13.9±16.08	1.16	0.0-57.7	0.162

Positive (n=24); Negative (n=21); NRS, Neonate Rectal Swab; %, Relative abundance of each taxon; SD, Standard Deviation; CV, Coefficient of Variation; *Range, denotes the minimum and maximum values in the relative abundance for each taxon; ***p*-value was calculated according to T-test; $p < 0.05$ are considered statistically significant differences.

Table S7. Diversity index of bacterial grouped positive and negative by RT-ddPCR.

Index	HC (-)	HC (+)	*p**	MRS (-)	MRS (+)	*p**	NRS (-)	NRS (+)	*p**
Observed	64.32±34.62	69.10±36.01	0.643	93.65±22.10	99.86±27.19	0.408	56.67±38.01	58.92±37.78	0.843
Chao 1	87.37±51.02	90.49±47.28	0.826	80.08±52.05	141.91±43.98	0.531	80.08±52.05	74.82±52.42	0.738
Shannon	2.23±1.14	2.34±1.02	0.729	3.18±0.76	3.13±0.72	0.816	2.05±1.13	2.45±1.08	0.242
Simpson	0.67±0.25	0.71±0.25	0.570	0.87±0.18	0.87±0.14	0.955	0.66±0.24	0.77±0.24	0.181

HC, Human Colostrum; MRS, Mother Rectal Swab; NRS, Neonatal Rectal Swab; Standard deviation is shown as ± values; **p*-value was calculated according to the T-test; $p < 0.05$ are considered a statistically significant difference.

International Journal of *Molecular Sciences*

MDPI

Table S8. Linear discriminant analysis (LDA) effect size (LEfSe) analysis, samples grouped according to SARS-CoV-2 rectal swab RT-ddPCR detection.

Group	Phylum	Taxa	LDA	*p*-value	*q*-value
MRS -	Actinobacteria	*g_Dietzia*	2.140	0.033	0.041
	Actinobacteria	*g_Microbacterium*	3.665	0.019	0.041
	Bacteroidetes	*g_Alloprevotella*	3.259	0.020	0.041
	Firmicutes	*g_Fenollaria*	3.614	0.036	0.041
	Firmicutes	*g_Granulicatella*	2.181	0.031	0.041
	Firmicutes	*g_Mogibacterium*	2.311	0.038	0.041
	Firmicutes	*g_Parvimonas*	2.207	0.038	0.041
	Firmicutes	*g_Saccharofermentans*	2.309	0.016	0.041
	Proteobacteria	*g_Bradyrhizobium*	3.385	0.027	0.041
	Proteobacteria	*g_Lautropia*	2.864	0.038	0.041
MRS +	Actinobacteria	*g_Coriobacteriaceae_UCG_3*	2.150	0.027	0.041
	Bacteroidetes	*g_Butyricimonas*	2.873	0.025	0.041
	Firmicutes	*g_Anaerotruncus*	2.245	0.021	0.041
	Firmicutes	*g_Candidatus_Soleaferrea*	2.276	0.030	0.041
	Firmicutes	*g_Clostridia_vadinBB6_group*	2.295	0.014	0.041
	Firmicutes	*g_Colidextribacter*	2.335	0.005	0.041
	Firmicutes	*g_Eubacterium_siraeum_group*	2.825	0.040	0.041
	Firmicutes	*g_Lachnospiraceae_ND3007_group*	2.456	0.022	0.041
	Firmicutes	*g_NK4A214_group*	2.860	0.039	0.041
	Firmicutes	*g_Oscillibacter*	2.804	0.036	0.041
	Firmicutes	*f_Ruminococcaceae*	2.172	0.027	0.041
	Firmicutes	*f_Ruminococcaceae_g_uncultured*	3.014	0.006	0.041
	Firmicutes	*g_UBA1819*	2.578	0.019	0.041
	Firmicutes	*g_UCG_10*	2.658	0.033	0.041
	Firmicutes	*g_Flavonifractor*	2.699	0.039	0.041
	Proteobacteria	*g_Undibacterium*	2.507	0.027	0.041
	Verrucomicrobiota	*g_Akkermansia*	2.825	0.049	0.049
NRS -	Actinobacteria	*g_Brevibacterium*	2.601	0.002	0.046
	Actinobacteria	*f_Cellulomonadaceae*	2.551	0.022	0.046
	Actinobacteria	*g_Nocardioides*	2.771	0.014	0.046
	Actinobacteria	*f_Propionibacteriaceae*	2.660	0.022	0.046
	Bacteroidetes	*g_Empedobacter*	2.693	0.022	0.046
	Firmicutes	*g_Aerococcus*	2.328	0.043	0.047
	Firmicutes	*g_Salinicoccus*	2.823	0.043	0.047
	Proteobacteria	*g_Allorhizobium*	2.771	0.043	0.047
	Proteobacteria	*g_Methylobacterium*	3.250	0.024	0.046
	Proteobacteria	*g_Pantoea*	2.629	0.043	0.047
	Proteobacteria	*g_Tepidimonas*	2.794	0.022	0.046
NRS +	Actinobacteria	*g_Bifidobacterium*	4.100	0.041	0.047
	Actinobacteria	*g_Libanicoccus*	2.745	0.042	0.047
	Firmicutes	*f_Christensenellaceae_R_7_group*	3.393	0.023	0.046
	Firmicutes	*g_Colidextribacter*	2.528	0.035	0.047
	Firmicutes	*g_Coprococcus*	2.658	0.035	0.047
	Firmicutes	*g_Granulicatella*	2.553	0.037	0.047
	Firmicutes	*f_Lachnospiraceae*	4.212	0.022	0.046
	Firmicutes	*g_Paraclostridium*	3.207	0.007	0.046
	Firmicutes	*g_Phascolarctobacterium*	2.564	0.010	0.046
	Firmicutes	*g_Subdoligranulum*	3.707	0.045	0.048
	Proteobacteria	*g_Ralstonia*	2.702	0.019	0.046
	Proteobacteria	*g_Undibacterium*	3.876	0.049	0.049
HC -	Actinobacteria	*f_Intrasporangiaceae*	2.496	0.039	0.046

	Firmicutes	*g_Chryseomicrobium*	2.258	0.027	0.046
	Firmicutes	*g_Gemella*	2.803	0.038	0.046
	Firmicutes	*g_Skermanella*	2.839	0.022	0.046
HC +	Actinobacteria	*g__Lawsonella*	3.031	0.018	0.046
	Cyanobacteria	*g_Sericytochromatia*	2.429	0.046	0.046
	Firmicutes	*f_Ruminoccocaceae, g_Incertae_Sedis*	2.257	0.046	0.046
	Firmicutes	*g__Monoglobus*	2.118	0.046	0.046
	Firmicutes	*f_Oscillospiraceae*	2.103	0.046	0.046
	Firmicutes	*g_Peptoniphilus*	2.933	0.028	0.046
	Proteobacteria	*g_Comamonas*	2.299	0.007	0.046
	Proteobacteria	*g_Serratia*	2.735	0.046	0.046

The threshold on the logarithmic LDA score was set to 2.0. p and q values < 0.05 were considered statistically significant. p-value was adjusted with the Benjamini-Hochberg method and generated FDR value (q-value). MRS (Mother Rectal Swab); NRS (Neonatal Rectal Swab); HC (Human Colostrum); f (family); g (genus); LDA (Linear Discriminant Analysis).

Table S9. Relative abundance at genera level of most common bacterial taxa found in MRS samples classified by qiime SourceTracker analysis.

Genera (MRS)	Abundance (% ± SD)	Genera (Unknow)	Abundance (% ± SD)
Acinetobacter	3.187±3.56	*Acinetobacter*	2.515±2.97
Bifidobacterium	0.594±1.50	*Allorhizobium*	1.038±6.81
Blautia	0.697±1.29	*Anaerococcus*	0.522±1.26
Bradyrhizobium	3.742±5.13	*Bradyrhizobium*	1.054±2.79
Clostridium	1.021±1.33	*Corynebacterium*	3.040±4.85
Collinsella	0.618±1.25	*Cutibacterium*	4.661±5.11
Dialister	0.661±1.65	*Dialister*	1.028±4.75
Enterobacter	0.556±2.43	*Escherichia*	2.017±14.14
Enterococcus	1.206±2.99	*Lactobacillus*	4.334±12.40
Escherichia	11.332±19.39	*Meiothermus*	0.950±1.24
Faecalibacterium	1.110±2.42	*Mesorhizobium*	0.527±0.96
Lactobacillus	1.969±2.91	*Micrococcus*	0.746±1.71
Microbacterium	7.286±8.11	*Porphyromonas*	0.550±3.51
Prevotella	5.331±14.99	*Prevotella*	1.803±6.07
Sphingomonas	3.634±3.88	*Proteus*	1.462±8.53
Staphylococcus	46.037±30.65	*Pseudomonas*	2.358±6.88
Streptococcus	6.308±12.13	*Sphingomonas*	0.927±1.11
Subdoligranulum	0.645±1.20	*Staphylococcus*	47.205±34.70
Other	3.560±6.09	*Streptococcus*	2.081±5.52
		Variovorax	1.253±3.45
		Veillonella	0.659±2.32
		Other	14.896±13.24

%, Relative abundance of each taxon; SD, Standard Deviation

International Journal of *Molecular Sciences*

MDPI

Figures

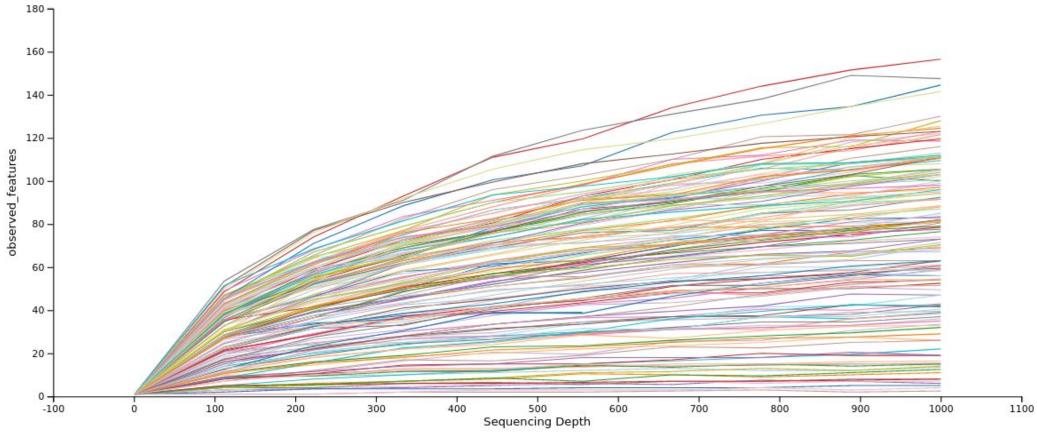

Figure S1. Rarefaction curves showing the bacterial richness based on observed features at 97% similarity using the Silva 138 database (Accessed in July 2022). Plots show total samples of MRS (Mother Rectal Swab), HC (Human Colostrum), and NRS (Neonate Rectal Swab) groups.

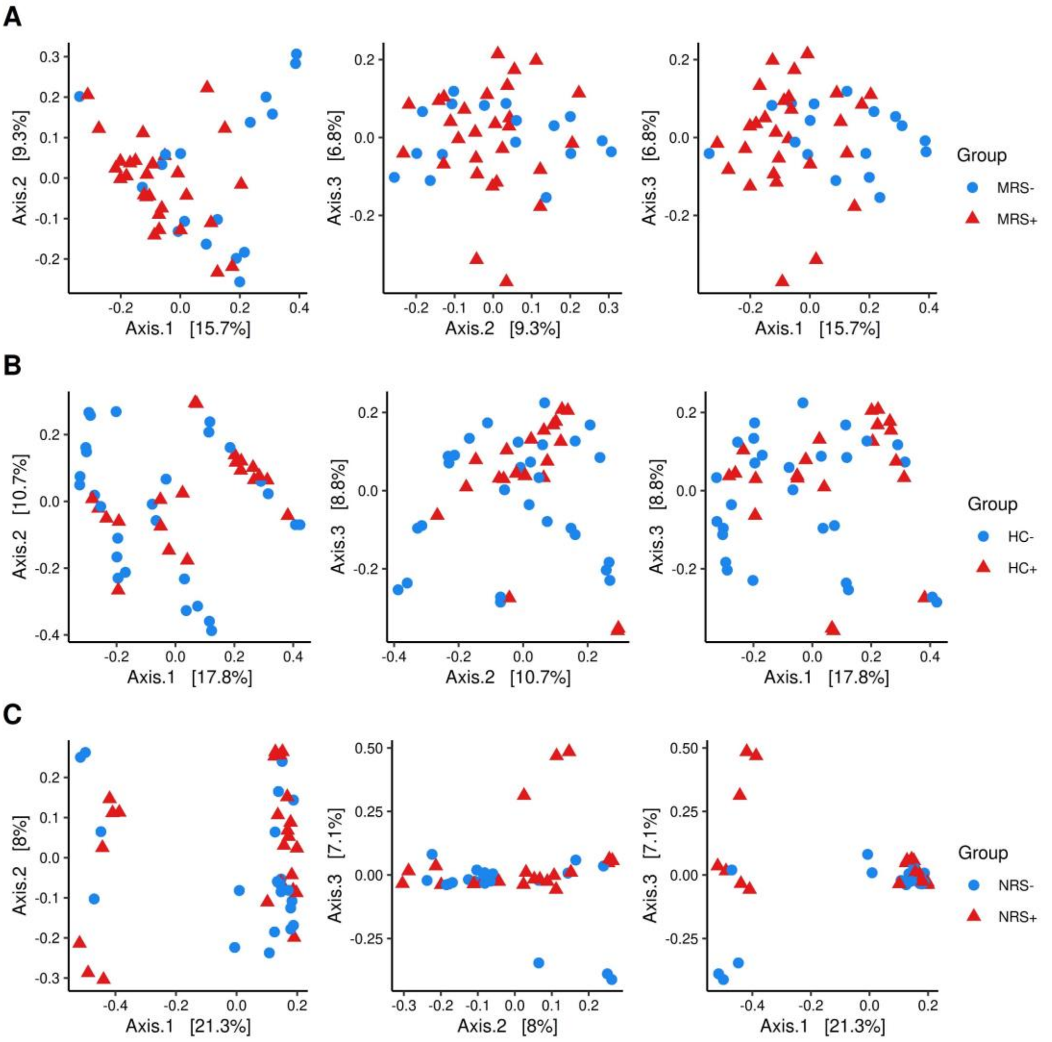

Figure S2. Beta diversity of bacteria in (**A**) MRS (Maternal Rectal Swab), (**B**) HC (Human Colostrum, and (**C**) NRS (Neonate Rectal Swab). The graphics show beta-diversity analyses calculated by dissimilarity metrics using the feature tables and Unweighted UniFrac analyses. The scatter plots were generated using principal coordinates analyses (PCoA) in three different axes showing the percentage of total differences. The positive (+) or negative (-) SARS-CoV-2 genome detection by ddPCR is shown beside the type of labels identified the samples at right side of the graphics. MRS group showed statistical differences according to ANOSIM (*p*-value = 0.004), HC and NRS got *p*-values of 0.105 and 0.175, respectively.

---end-of-document—
